# Mitochondrial DNA as a target for analyzing the biodistribution of cell therapy products

**DOI:** 10.1038/s41598-024-56591-4

**Published:** 2024-04-04

**Authors:** Young-Woo Cho, Jaehyeon Yoon, Suk-Gil Song, Young-Woock Noh

**Affiliations:** 1 College of Pharmacy, Chungbuk National University, Cheongju, 28644 Republic of Korea; 2 Division of Drug Screening Evaluation, NDDC, Osong Medical Innovation Foundation, Cheongju, 28160 Republic of Korea

**Keywords:** Cell therapy product, Biodistribution, Mitochondrial DNA, *Alu* repeat, Medical research, Preclinical research

## Abstract

Biodistribution tests are crucial for evaluating the safety of cell therapy (CT) products in order to prevent unwanted organ homing of these products in patients. Quantitative polymerase chain reaction (qPCR) using intronic *Alu* is a popular method for biodistribution testing owing to its ability to detect donor cells without modifying CT products and low detection limit. However, *Alu*-qPCR may generate inaccurate information owing to background signals caused by the mixing of human genomic DNA with that of experimental animals. The aim of this study was to develop a test method that is more specific and sensitive than *Alu*-qPCR, targeting the mitochondrial DNA (mtDNA) sequence that varies substantially between humans and experimental animals. We designed primers for 12S, 16S, and cytochrome B in mtDNA regions, assessed their specificity and sensitivity, and selected primers and probes for the 12S region. Human adipose-derived stem cells, used as CT products, were injected into the tail vein of athymic NCr-nu/nu mice and detected, 7 d after administration, in their lungs at an average concentration of 2.22 ± 0.69 pg/μg mouse DNA, whereas *Alu* was not detected. Therefore, mtDNA is more specific and sensitive than *Alu* and is a useful target for evaluating CT product biodistribution.

## Introduction

The biodistribution of cell therapy (CT) products is an important factor to consider in predicting and assessing the efficacy and toxicological profiles of these products in nonclinical and clinical studies^1,2^. Biodistribution studies of CT products have been conducted using real-time (RT) polymerase chain reaction (PCR), labeling with dyes, genetic modification (using green fluorescent protein or luciferase), radioactive isotopes, and labeling with nanoparticles^1,3–6^. Compared to other methods, RT- PCR has several advantages, including its ability to identify donor cells without modifying CT products and its high sensitivity^3,7–9^.

Recently, human DNA-specific PCR probes, such as intronic *Alu* repeats, have been used to identify CT products^7,10^. *Alu* repeats are primate-specific short interspersed elements (SINEs) that account for 10% of the human genome^10,11^ and are useful targets for detecting human cells. However, when mixed with genomic DNA (gDNA) from experimental animals, they produce background signals, resulting in the generation of inaccurate information^10^. This phenomenon can be attributed to nonspecific reactions caused by *Alu*-related 7SL RNA-derived SINEs or B1, the presence of multiple target sites owing to repetitive sequences, and the close proximity of *Alu* pairs, because of which one primer can reach the next *Alu* sequence and amplify the region^12^. The detection limit of *Alu*-based quantitative PCR (qPCR) in purified human gDNA is approximately 10 femtograms (fg); however, during gDNA contamination, it is reportedly in the order of picograms^7,13,14^.

Mitochondrial DNA (mtDNA), which is circular and double-stranded, is a reliable tool for species identification owing to its higher degree of variability among different species relative to that of nuclear DNA^15,16^. Therefore, primers designed for the amplification of specific human mtDNA segments may not amplify segments corresponding to mtDNA segments from other species. Therefore, mtDNA is a useful target for distinguishing human cells from those of experimental animals, making them suitable for application in studies on the biodistribution of CT products^17^. However, the utility of mtDNA compared to *Alu*-qPCR in distinguishing human DNA from the DNA of experimental animals and its facilitation of quantification have not been explored. As mtDNA is longer than the *Alu* element and shows considerable differences among animal species, it is advantageous for target selection. In addition, mtDNA accounts for a large proportion of total DNA; thus, a quantitative method using RT-PCR based on mtDNA can be developed. Therefore, the aim of this study was to investigate the utility of mtDNA in distinguishing human and animal cells, using specific primers and probes.

## Results

### Comparison of *Alu*-qPCR results among different species

**Figure 1:**
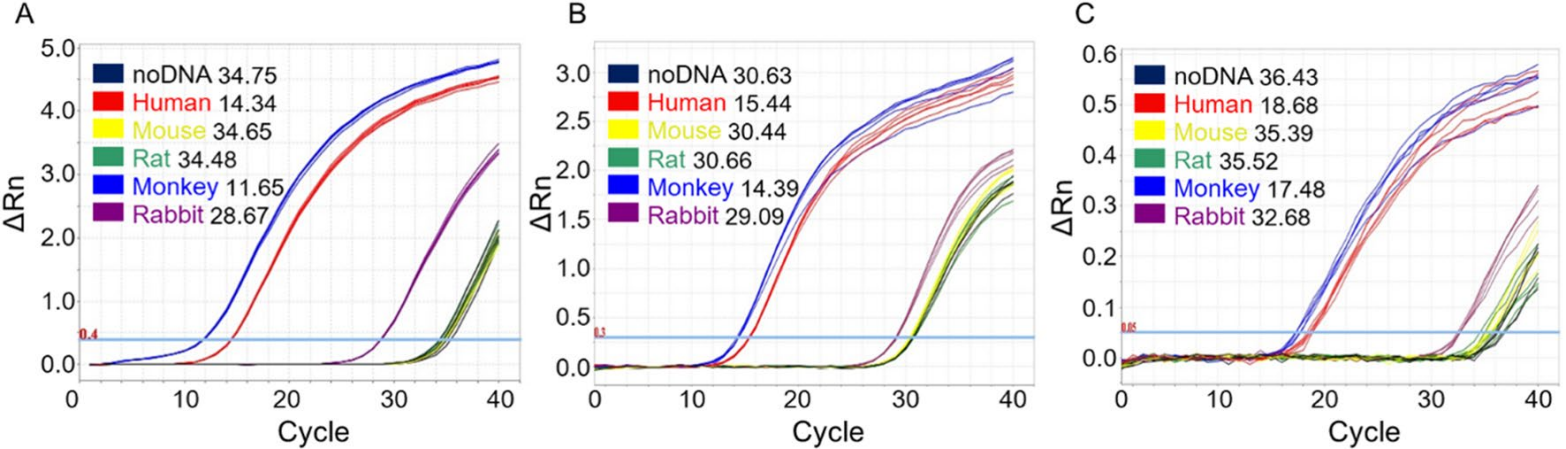
HYPERLINK "sps:id::fig1||locator::gr1||MediaObject::0"Comparison of *Alu*-qPCR performance among different species. The background signal of *Alu*-qPCR was confirmed in the analysis of DNA of experimental animals. (**A**) represents the primer/probe used in this study, (**B**) is the result when using the primer/probe by McBride et al., and (**C**) is the result when using the primer/probe by Funakoshi et al. For humans (red) and monkeys (blue), 5 ng of gDNA was used. For mice (yellow), rats (green), and rabbits (purple), 1 μg of gDNA was used. qPCR was performed in triplicates in a single run. Ct values were analyzed using ABI 7500 software v.2.3. The threshold was set at 0.4 (in red), and the light blue line in the graph indicates the threshold across all study participants. gDNA, genomic DNA; mtDNA, mitochondrial DNA; qPCR, quantitative polymerase chain reaction.

We conducted a thorough investigation of background signals arising from potential contamination of laboratory animal gDNA using the *Alu*-qPCR system (Fig. 1). Three primer and probe sets were used (A: This study, B: McBride et al.^18^, C: Funakoshi et al.^10^), and the result patterns were found to be similar. The obtained cycle threshold (Ct) values for 1 µg of mouse were A: 34.65, B: 30.44 and C: 35.39, respectively. For rat DNA, the Ct values were identified as A: 34.48, B: 30.66 and C: 35.52, respectively. For rabbit DNA, the Ct values were identified as A: 28.67, B: 29.09 and C: 32.68, suggesting the presence of nonspecific amplification as a background signal. Notably, the amplification of monkey DNA was not considered a nonspecific reaction owing to the presence of *Alu* repeat sequences in the primate genome. These findings provide crucial insights into the potential sources of background signals in our experimental setup.

### Selection of primers and probes for mtDNA

**Figure 2:**
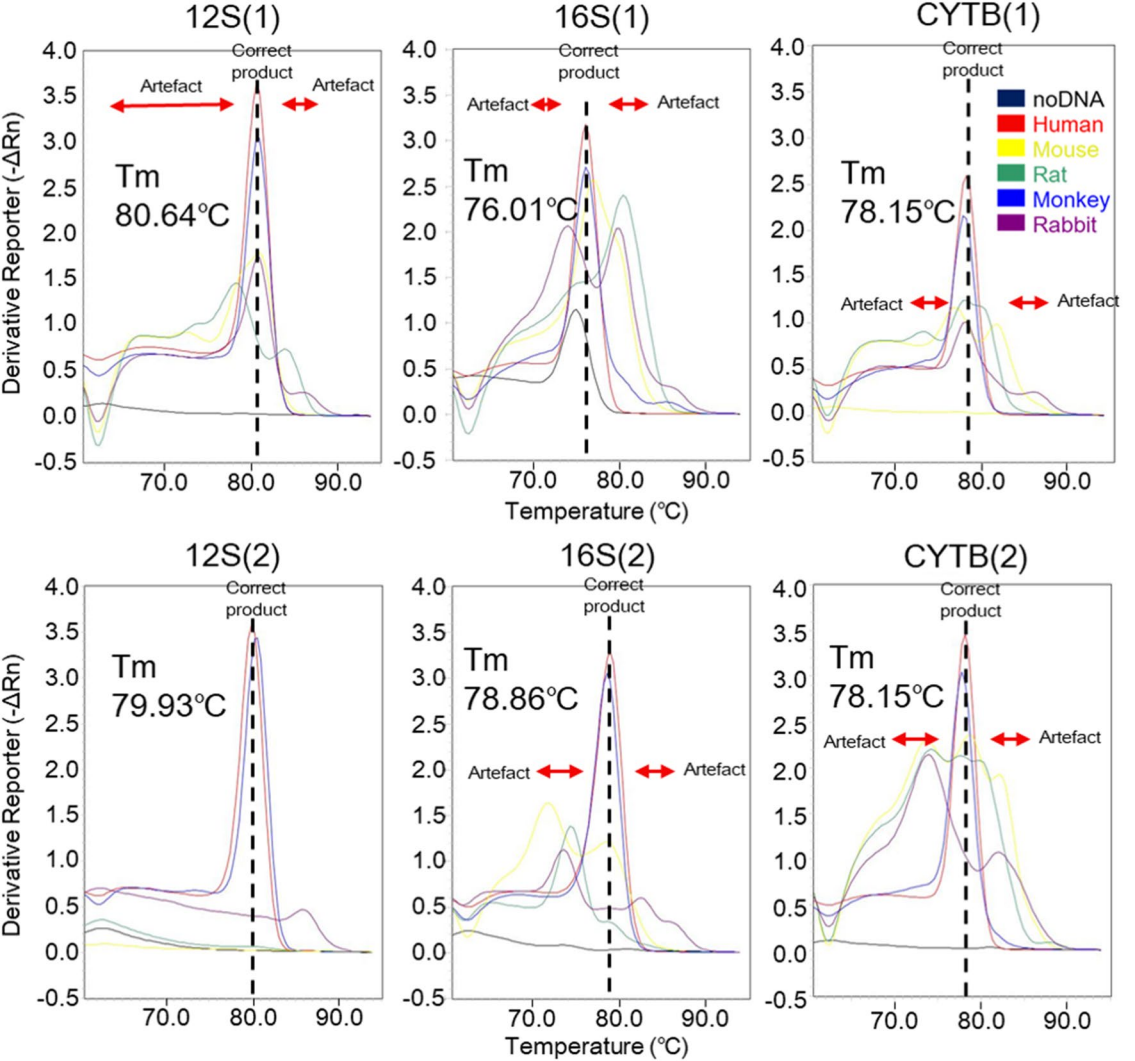
Confirmation of nonspecific reaction using primer candidates during mitochondrial DNA detection. Linear approximation (black line) indicates the melting temperature (Tm) value for human DNA (red line). The Tm value estimated as an artifact is indicated by the red arrow.

The selection of appropriate primer and probe sets for mtDNA amplification was conducted using six primer pairs targeting three different regions of mtDNA: 12S rRNA, 16S rRNA, and cytochrome B. Among these primer pairs, the 12S rRNA (2) pair exhibited a Ct value above 40 in rodents, and no nonspecific reactions were observed (Table 1). In contrast, the other five primer pairs exhibited relatively low or similar Ct values compared to those of existing *Alu* primers, which ranged between 28.25 and 36.96. We confirmed a single melting temperature (Tm) of human DNA for all six primer pairs. However, for the DNA of the experimental animals, we observed two to three Tms for all primer pairs, except for the 12S rRNA (2) pair (Fig. 2). After careful consideration, we selected the 12S rRNA (2) pair, which did not nonspecifically react with the DNA of laboratory animals, as our final candidate.

**Table 1:** Quantitative ability and specificity of primer candidates for mitochondrial DNA.

	12S(1)				**12S(2)**				16S(1)				16S(2)				*CYTB*(1)				*CYTB*(2)			
	Ct	Tm1	Tm2	Tm3	Ct	Tm1	Tm2	Tm3	Ct	Tm1	Tm2	Tm3	Ct	Tm1	Tm2	Tm3	Ct	Tm1	Tm2	Tm3	Ct	Tm1	Tm2	Tm3
Blank (0 ng)	< 40*	–	–	–	< 40*	**–**	**–**	**–**	37.03	74.77	–	–	< 40*	–	–	–	< 40*				< 40*			
Human (5 ng)	16.66	80.64	–	–	**17.14**	**79.93**	**–**	**–**	17.32	76.01	–	–	17.29	78.86	–	–	16.93	78.15	–	–	16.78	78.15	–	–
Mouse (1000 ng)	33.52	81.00	–	–	< 40*	**80.29**	**–**	**–**	28.25	76.90	–	–	33.14	71.74	78.51	–	34.70	76.90	81.53	68.71	31.44	78.69	73.70	82.07
Rat (1000 ng)	35.09	78.33	84.03	–	< 40*	**62.48**	**–**	**–**	29.46	80.29	–	–	36.96	74.41	–	–	35.53	77.97	73.17	–	33.38	74.23	77.62	
Monkey (1000 ng)	24.05	80.82	–	–	**24.75**	**80.47**	**–**	**–**	23.47	76.01	–	–	24.65	78.51	–	–	24.94	77.97	–	–	24.96	77.80	–	–
Rabbit (1000 ng)	32.81	80.82	–	–	**37.05**	**85.81**	**62.30**	**–**	29.11	73.88	79.75	–	36.95	73.52	82.43	–	33.92	77.97	–	–	32.00	73.88	82.07	

*< 40, Not amplified up to 40 cycles; Ct, cycle threshold; Tm, melting temperature. Bold, final candidate for mitochondrial DNA quantitative PCR.

### Comparison of qPCR using *Alu* and mtDNA

**Figure 3:**
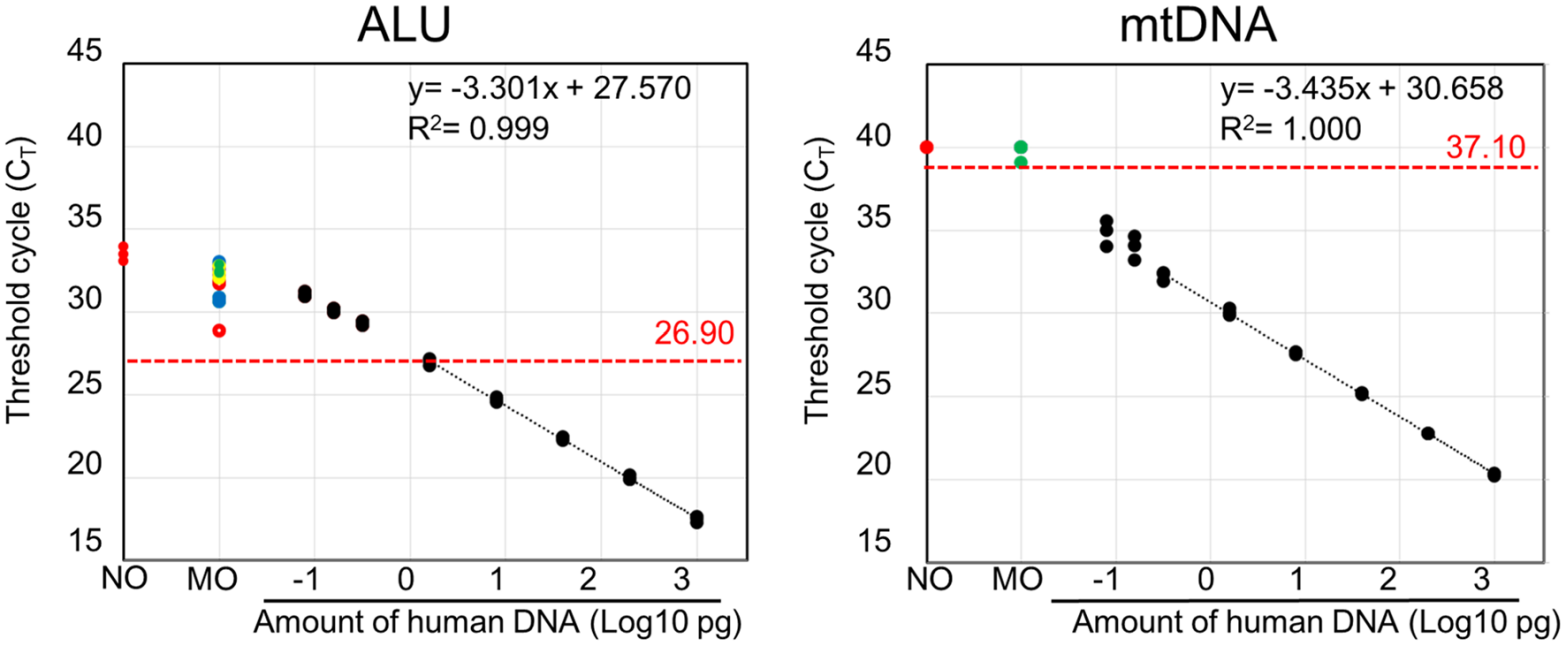
Standard curve for detecting human gDNA using primer and probe sets for *Alu* (**A**) and mtDNA (**B**). qPCR was performed in triplicate in a single run, and mean Ct values were plotted. In cases where samples were not detected, a Ct value of 40 was assumed. Negative control samples contained mouse gDNA only (brain, yellow dot; heart, green dot; lung, blue hollow circle; liver, orange dot; kidney, purple dot; spleen, red hollow circle; and pancreas, blue dot) and were used as NTCs (red dots). Detection threshold was established as two cycles below the lower Ct value of the the mouse DNA or noDNA (indicated by the red and dotted lines), and the corresponding number of cycles is highlighted in red. The linear approximation equations (black lines) and their corresponding R^2^ values are also displayed. Although not detected, a dot was placed at the 40-cycle mark to ease visualization. gDNA, genomic DNA; mtDNA, mitochondrial DNA; qPCR, quantitative polymerase chain reaction; MO, mouse DNA; NO, noDNA.

The sensitivity of primers and probes was assessed using different concentrations of human gDNA, ranging from 0.08 to 1000 pg. For comparison, we examined the *Alu* primer–probe set shown in Fig. 1. The lower limit of quantification (LLOQ) was determined as the minimum value that satisfied the following two conditions (Table 2): the minimum concentration at which accuracy and precision met the criteria for the limit of quantification (accuracy, 80%–120%; coefficient of variation [CV], ± 20%), and minimum concentration lower than the Ct value of the mouse DNA and noDNA. Thus, the limits of quantification were 8.0 and 0.32 pg for *Alu* and mtDNA, respectively (Fig. 3). The lower limit of quantification of Alu-qPCR as presented by McBride et al. and Funakoshi et al. were confirmed to be 8.0 and 1.6 pg, respectively (Supplementary Fig. S4). The results for *Alu* showed differences among organs in the mouse DNA (Fig. 3A). Furthermore, the amplification observed for the mouse DNA and noDNA samples could be attributed to nonspecific reactions between the primers and probes. When these nonspecific reactions are identified close to the limit of detection, they may complicate the interpretation of the results. These findings indicate that if the differences in nonspecific responses for each tissue increase, the limit of quantification may also increase. In contrast, among the six tissue samples analyzed in triplicates (Fig. 3B), the Ct value of mtDNA of only one sample was found to be 39.10. The detection limit was arbitrarily set based on a Ct value two cycles below that of the mouse DNA and noDNA. Furthermore, the detection limits for *Alu* and mtDNA were 1.596 and 0.013 pg, respectively. The detection limits of Alu-qPCR as presented by McBride et al. and Funakoshi et al. were confirmed to be 2.12 and 0.70 pg, respectively (Supplementary Fig. S4). Therefore, compared to *Alu*-qPCR, mtDNA-qPCR demonstrated a fivefold improvement in the LLOQ and a remarkable 54-fold enhancement in the limit of the detection (LOD), indicating that the mtDNA-qPCR assay detected a lower amount of target DNA than the *Alu*-qPCR assay. These results highlight the superior sensitivity and detection capability of the mtDNA-qPCR method over the *Alu*-qPCR approach.

**Table 2:** Lower limit of quantification and limit of detection for human adipose-derived stem cells.

*Alu*	Human DNA (pg)							
	STD6	STD5	STD4	STD3	STD2	STD1	LOD1	LOD2
	1000.00	200.00	40.00	8.00	1.60	0.32	0.16	0.08
Ct mean	17.49	19.99	22.38	24.71	26.99	29.33	30.10	31.02
Ct s.d.	0.15	0.12	0.08	0.11	0.17	0.10	0.11	0.12
Mean concentration	1138.05	198.18	37.42	7.38	1.51	0.29	0.17	0.09
s.d.	119.32	15.64	2.19	0.57	0.18	0.02	0.01	0.01
Accuracy (%)	113.81	99.09	93.55	92.28	94.52	92.01	107.71	112.88
CV (%)	10.48	7.89	5.86	7.73	11.69	7.31	7.94	8.23

mtDNA	Human DNA (pg)							
	STD6	STD5	STD4	STD3	STD2	STD1	LOD1	LOD2
	1000.00	200.00	40.00	8.00	1.60	0.32	0.16	0.08
Ct mean	20.30	22.75	25.17	27.63	30.05	32.24	33.96	34.84
Ct s.d.	0.06	0.02	0.04	0.08	0.16	0.22	0.58	0.64
Mean concentration	1,040.19	199.98	39.64	7.64	1.52	0.35	0.12	0.07
s.d.	39.35	2.42	1.07	0.44	0.16	0.05	0.05	0.03
Accuracy (%)	104.02	99.99	99.09	95.46	94.70	109.77	73.58	83.12
CV (%)	3.78	1.21	2.69	5.75	10.81	15.45	39.15	44.44

The STD and LOD are prepared by performing a 1/5 serial dilution of DNA isolated from hADSC. In this case, the concentration is based on the DNA concentration of hADSC.

STD, standard; s.d., standard deviation; Ct, cycle threshold; mtDNA, mitochondrial DNA; CV, coefficient of variation.

### Biodistribution of hADSCs after administration in mice

Both assays using *Alu* and mtDNA were suitable for detecting hADSCs (Supplementary Tables S1–S5). *Alu* or mtDNA was not detected in the vehicle control groups. However, 1 h after hADSC administration, both *Alu* and mtDNA were detected in the lungs of all animals, with partial detection in the liver of one animal. No cells were detected in the brain, heart, kidneys, spleen, testes, pancreas, or bone marrow. *Alu* was detected in the lungs at an average of 11.47 ± 6.44 pg/μg mouse DNA, whereas mtDNA was detected in the lungs at an average of 23.19 ± 4.68 pg/μg mouse DNA.

**Figure 4:**
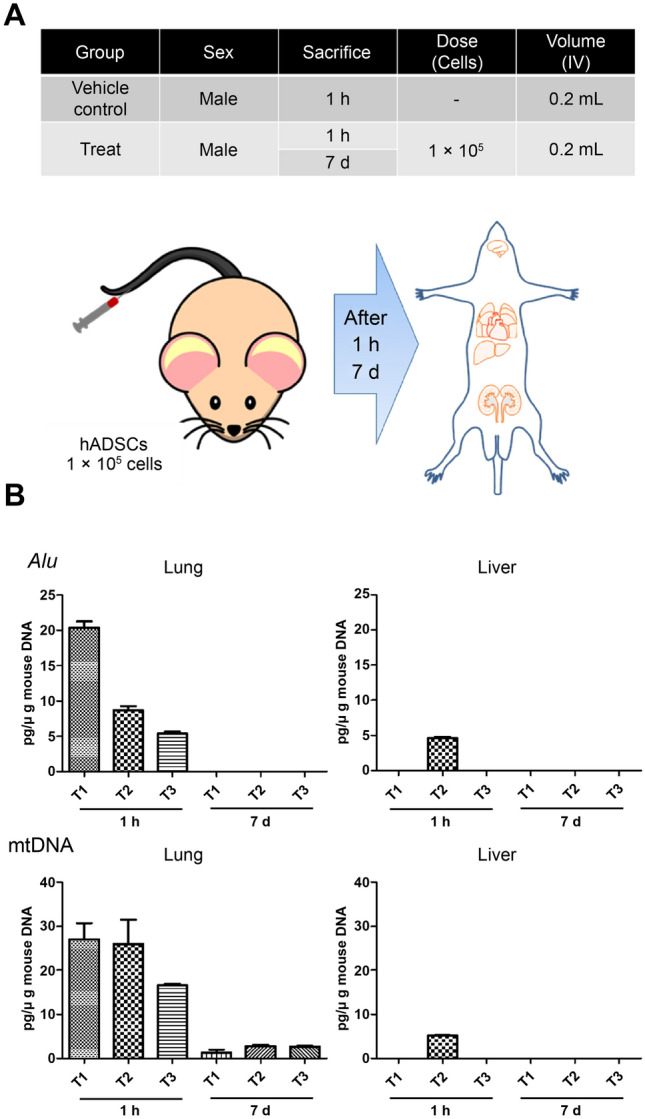
Detection of human cells in different organs 1 h and 7 d after the injection of hADSCs into the tail vein of BALB/c nude mice. (**A**) Summary and schematic of animal experiments. (**B**) Comparison of results between *Alu*-qPCR and mtDNA-qPCR. qPCR was performed in triplicates in a single run. Results of organs in which hADSCs were not detected are excluded from the graph. The data presented represent mean and the bars indicate standard deviation for n = 3. mtDNA, mitochondrial DNA; qPCR, quantitative polymerase chain reaction; hADSC, human adipose-derived stem cell.

Seven days after hADSC administration, mtDNA was detected in the lungs of all animals; however, *Alu* was not detected. No hADSCs were detected in the brain, heart, liver, kidneys, spleen, testes, pancreas, or bone marrow. Moreover, mtDNA was detected in the lungs at an average of 2.22 ± 0.69 pg/μg mouse DNA, which was lower than that at the earlier time point (Fig. 4). Overall, these results suggest that the mtDNA-qPCR assay has a higher sensitivity and lower limits of quantification and detection than the *Alu*-qPCR assay.

## Discussion

In this study, we developed a highly sensitive and specific mtDNA-qPCR method for detecting human gDNA mixed with gDNA of laboratory animals. For this purpose, we compared the mtDNA sequences of humans and experimental animals and identified a sensitive and specific target that did not engage in nonspecific reactions. The *Alu*-qPCR system showed background signals in the analysis of experimental animal samples.

Consistent with the results of previous investigations^7,10,19^, our study revealed nonspecific amplification of experimental animal samples by the *Alu*-qPCR system. Although the background signals generated with the use of the *Alu*-qPCR system have been observed in previous studies, they have not been highlighted as important factors. Creane et al*.* reported results similar to ours, with Ct values of 35.93 and 35.89 for the DNA of mice and rats, respectively^7^. Prigent et al*.* also reported nonspecific amplification with *Alu*Tb8 using 1 µg of mouse and rat DNA samples, and the Ct values obtained were 31.08 and 32.07, respectively^19^. Funakoshi et al*.* reported an improvement in the nonspecific amplification of *Alu* caused by the gDNA of laboratory animals using highly specific primers and probes^10^. In this study, we also used the primers and probes utilized by McBride et al. and Funakoshi et al. to conduct experiments under the same conditions. According to Funakoshi et al., the Alu was detected in mouse DNA only after more than 40 cycles and was not detected in the noDNA control group. However, in our study, it was detected in shorter cycles. This difference could potentially be due to various factors such as genetic variations in mice, breeding conditions, experimental conditions, or the experimental equipment used.

In the present study, differences in background signals were observed for each organ and sample of mouse DNA and noDNA when the *Alu*-qPCR system was used. In biodistribution testing, it is common to evaluate rodents DNA using one or two representative samples. However, our experimental findings highlighted the importance of evaluating each experimental animal and organ individually when validating biodistribution test results obtained using the *Alu*-qPCR system. Therefore, as our mtDNA-qPCR method did not exhibit nonspecific reactions in animal organ DNA, it is more advantageous than the *Alu*-qPCR system in terms of specificity and reliability for biodistribution studies.

In this study, we conducted experimental investigations to demonstrate the suitability of mtDNA as a valuable target for analyzing human cells. The high copy number of mtDNA and substantial sequence variations across species make it an attractive choice for such analyses. By leveraging these unique characteristics of mtDNA, we can gain valuable insights into the molecular composition and genetic diversity of human cell populations. However, it should be noted that we did observe some instances of nonspecific amplification in certain experimental animals, despite our efforts. Although we designed six primer pairs targeting specific regions within three mtDNA genes, only one pair did not show evidence of nonspecific amplification with DNA from the experimental animals. The Ct values obtained for rodents ranged from 28.25 to 35.53 (Table 1), comparable to the results obtained using the *Alu*-qPCR system. A previous study established that mitochondrial 12S rRNA and 16S rRNA genes exhibit high conservation across a wide range of organisms, such as fish, amphibians, and mammals, including humans^20^. However, our analysis revealed that the substantial differences observed between species occur only in a small portion of these genes (Supplementary Figs. S1–S3), making it challenging to design target regions within this limited area. These findings suggest the need for careful primer design and optimization to minimize nonspecific amplification and improve the specificity of mtDNA-based assays.

Biodistribution studies using mtDNA-qPCR have some limitations. The copy number of mtDNA varies depending on several factors, including diseases, aging, and oxidative stress^21–24^. The ratio of nuclear DNA to mtDNA may also vary within an organism because the number of mitochondria in a cell may change depending on the cell type and physiological state of the organism. These factors can affect the accuracy and precision of mtDNA-qPCR for biodistribution studies. Thus, it is paramount to carefully consider these limitations when interpreting the findings derived from mtDNA-qPCR-based biodistribution investigations. Nevertheless, it should be noted that in biodistribution studies involving CT products, the analysis typically involves cells manufactured following the good manufacturing practice (GMP) or GMP-like standards, which include comprehensive process development prior to their application in phase 1 clinical trials. Furthermore, given the stringent quality control measures implemented during the production of CT products, substantial changes in mtDNA contents are not expected in the final products, as the cell state and purity are rigorously ensured.

In the present study, in in vivo experiments, the mtDNA-qPCR system detected human gDNA in the lungs of mice, even after 7 d of intravenous injection of hADSCs, whereas the *Alu*-qPCR system failed to detect human gDNA, probably owing to the nonspecific background signal of the *Alu*-qPCR system. These findings strongly suggest that the mtDNA-qPCR system developed in this study is a reliable and specific method for the in vivo detection of human gDNA. This approach holds promise for the accurate identification and characterization of cells from different species, further expanding the applications of mtDNA-based detection methods in the field of cell biology and regenerative medicine. We anticipate that the mtDNA-qPCR method will make a valuable contribution to preclinical studies, particularly in the sensitive and specific detection of genetic substances within the human genome.

## Materials and methods

### Cell culture

We selected hADSCs among stem cells that are frequently used for developing CT products. These cells were cultured to obtain human DNA for preparation of standard template DNA for Alu-qPCR and mtDNA-qPCR and were also used for in vivo administration. To culture STEMPRO Human Adipose-Derived Stem Cells (Thermo Fisher Scientific, Waltham, MA), the MesenPRO RS Medium Kit (Thermo Fisher Scientific, Waltham, MA) and l-glutamine (Thermo Fisher Scientific, Waltham, MA) were used according to the manufacturer's instructions. The cells were cultured in 75-cm^2^ flasks at 37 °C and 5% CO_2_.

### Preparation of standard template DNA

The standard template DNA for the calibration curve was extracted hADSC. The extracted gDNA was quantified using a NanoDrop UV–Vis Spectrophotometer (Thermo Fisher Scientific, Waltham, MA, and then diluted with nuclease-free water at a 1/5 ratio (concentrations: 1000, 200, 40, 8, 1.6, 0.32, 0.16, and 0.08 pg). For qPCR analysis, this standard sample was spiked with 1µg of gDNA extracted from a mouse to create a quantification curve.

### Primer and probe design

To identify the mtDNA sequence that amplifies only human DNA, we used ClustalW v.7.0.5.3 in BioEdit^25^ to identify two ribosomal RNA genes (12S and 16S) and cytochrome b (*CYTB*) gene. We aligned and compared these sequences with those of humans, mice, rats, monkeys, and rabbits and identified the regions with the highest differences (Supplementary Figs. S1–S3). Thereafter, primers were designed for these regions using Primer3web v.0.4.0). The primers and probes were synthesized by Macrogen (Seoul, Republic of Korea) and Thermo Fisher Scientific (Waltham, MA). The primer sequences are listed in Table 3. The relevant parameters were as follows: primer size, 18–20 bp; Tm, 57–60 °C; GC ratio, 30–80%; and amplicon size, 50–180 bp. The probe parameters were as follows: size, 18–20 bp; Tm, 68–70 °C; and GC ratio, 30–80%.

**Table 3:** Primers and probes used in this study.

Name	Type^a^	Sequence
*Alu* (this study)	F	5′-ACCTGAGGTCAGGAGTTTGAGA-3′
	R	5′-GGTTCAAGCGATTCTCCTGC-3′
	P	5′-FAM-CAACATGGTGAAACCC-MGB-3′
*Alu2* (McBride et al.)	F	5′-CATGGTGAAACCCCGTCTCTA-3′
	R	5′-GCCTCAGCCTCCCGAGTAG-3′
	P	5′-FAM-ATTAGCCGGGCGTGGTGGCG-TAMRA-3′
*Alu3* (Funakoshi et al.)	F	5′-GGTGAAACCCCGTCTCTACT-3′
	R	5′-GGTTCAAGCGATTCTCCTGC-3′
	P	5′-FAM-CGCCCGGCTAATTTTTGTA-BHQ1-3′
12S (1)	F	5′-TCCCCGTTCCAGTGAGTTCA-3′
	R	5′-ATCACTGCTGTTTCCCGTGG-3′
12S (2)	F	5′-CGCAGCAATGCAGCTCAAAA-3′
	R	5′-ACGCCGGCTTCTATTGACTTG-3′
	P	5′-FAM-AGCCTAGCCACACCCCCACG-MGB-3′
16S (1)	F	5′-GGCCTAAAAGCAGCCACCAA-3′
	R	5′-TGGTCCAATTGGGTGTGAGG-3′
16S (2)	F	5′-ACGAGGGTTCAGCTGTCTCT-3′
	R	5′-GGACCTGTGGGTTTGTTAGGT-3′
*CYTB* (1)	F	5′-TCTCCGATCCGTCCCTAACA-3′
	R	5′-AGAATGAGGAGGTCTGCGGC-3′
*CYTB* (2)	F	5′-ACAATTCTCCGATCCGTCCCT-3′
	R	5′-ATGAGGAGGTCTGCGGCTAG-3′

^a^F, forward primer; R, reverse primer; P, probe.

### PCR

To confirm background amplification in *Alu*-qPCR, DNA from humans, mice, rats, monkeys, and rabbits was amplified (Fig. 1). The primers and probes for human *Alu* were used as candidates validated for biodistribution studies at the Osong Medical Innovation Foundation. Additionally, a comparison was made with the primers and probes used in the research by McBride et al. and Funakoshi et al. Furthermore, in this study, we developed an mtDNA-based qPCR method that is highly sensitive to human gDNA when human-derived cells are administered to animals. The primers and probes were designed to avoid the nonspecific binding of rodent gDNA during biodistribution studies. In addition, the mtDNA-qPCR method was more applicable relative to *Alu*-qPCR in terms of its specificity and relatively low limits of quantification and detection. For primer selection for mtDNA [12S (1), 12S (2), 16S (1), 16S (2), *CYBT* (1), and *CYBT* (2)], a 10-μL reaction mixture was prepared by adding 5 μL of SYBR Master Mix (Thermo Fisher Scientific), 0.2 μM of forward and reverse primers each, and an appropriate amount of gDNA. The reactions were then performed using an Applied Biosystems 7500 FAST RT PCR instrument (Thermo Fisher Scientific, Waltham, MA) with the following conditions: one cycle at 95 °C for 10 min, followed by 40 cycles at 95 °C for 15 s, and 60 °C for 1 min. Melting curves were obtained by gradually increasing the temperature of the system from 65 to 95 °C with a 1% increase in temperature every 2 s.

Moreover, qPCR for *Alu* and mtDNA with primers and probes were performed with a 10-μL reaction mixture containing FAST advanced Master Mix (Thermo Fisher Scientific, Waltham, MA), 0.2 μM of forward and reverse primers, and 0.25 μM of probe. The PCR conditions were as follows: 1 cycle at 50 °C for 2 min and 95 °C for 10 min, followed by 40 cycles at 95 °C for 15 s and 60 °C for 1 min. Standard curves were generated by adding fivefold serial dilutions of the gDNA of hADSCs to the gDNA of mice. Additionally, the primers and probes from McBride et al. (Alu2) and Funakoshi et al. (Alu3) were used for PCR under the conditions described in their research. The Alu2 qPCR was performed with a 10-μL reaction mixture containing TaqMan Universal Master Mix II, no UNG (Thermo Fisher Scientific, Waltham, MA), 0.9 μM of forward and reverse primers, and 0.25 μM of probe. The PCR conditions were as follows: 1 cycle at 50 °C for 2 min and 95 °C for 10 min, followed by 40 cycles at 95 °C for 15 s and 60 °C for 1 min. The Alu3 qPCR was performed with a 10-μL reaction mixture containing TaqMan Universal Master Mix II, no UNG (Thermo Fisher Scientific, Waltham, MA), 0.2 μM of forward and reverse primers, and 0.25 μM of probe. The PCR conditions were as follows: 1 cycle at 95 °C for 10 min, followed by 40 cycles at 95 °C for 15 s, 56 °C for 30 s, and 60 °C for 30 s. Standard curves were generated by adding fivefold serial dilutions of the gDNA of hADSCs to the gDNA of mice.

The performances of *Alu* and mtDNA were assessed by comparing their quantification ranges and detection limits. gDNA from different tissues (brain, heart, liver, pancreas, kidneys, and spleen) of mice was also extracted and analyzed using 1 μg of gDNA in triplicates as NTCs to detect background signals. The PCR data obtained were analyzed using ABI 7500 software v.2.3 (Thermo Fisher Scientific, Waltham, MA).

### Validation

We conducted a study to comprehensively validate the assay method for quantifying hADSCs in DNA isolated from the test animal tissues. The validation of the mtDNA-qPCR and *Alu*-qPCR methods for biodistribution analysis was configured as described below to evaluate the detection limit, linearity, accuracy, and precision**.**

#### Calibration range and LOD

To establish the range of the calibration curve and LLOQ, a standard sample was analyzed using qPCR. Thereafter, the calibration range and LLOQ were evaluated to determine whether the accuracy and precision satisfied the standards in the range within which linearity was confirmed. The tests were repeated three times, and the lowest concentration that satisfied the criteria for accuracy and precision was set as the LLOQ. The detection limit satisfied the following conditions:

- A concentration lower than LLOQ
- A concentration that yielded a Ct value that was clearly different from that of NTC (NTC Ct – 2 cycles).

Criteria: Accuracy within 100% ± 15% and LLOQ accuracy within 100% ± 20%

#### Linearity

The linearity of the calibration curve was analyzed via qPCR using a calibration standard prepared with different concentrations of standard template DNA. The calibration standard was used to prepare the calibration curve and calculate the coefficient of determination, y-intercept, and slope of the regression line. For the calibration curve, the blank (BLK), NTC, and six concentration ranges from the upper limit of quantitation to the LLOQ were analyzed three times with five wells for each concentration. We also verified whether linearity satisfied these criteria.

*Criteria: R^2^ ≥ 0.99, accuracy within 100% ± 15%, LLOQ accuracy within 100% ± 20%, CV within 15%, and LLOQ CV within 20%.

#### Accuracy

High-quality control (HQC), medium-quality control (MQC), low-quality control (LQC), and LLOQ were established based on the linearity results to evaluate accuracy. The experiments were performed in triplicates on two different days with four different quality control samples (HQC, MQC, LQC, and LLOQ) to verify the satisfaction of the accuracy criteria based on recovery. At least three samples were used for each QC analysis. Reproducibility was evaluated based on intra-day and inter-day accuracy.

* Criteria: Accuracy within 100% ± 15% and LLOQ accuracy within 100% ± 20%.

#### Precision

To determine precision, experiments were performed in triplicates using four different QC samples to verify whether the CV value satisfied the precision criteria. The experiments were repeated on different days. At least three samples were analyzed for each QC. Reproducibility was evaluated based on the precision of intra-day and inter-day experiments.

*Criteria: CV within 15% and LLOQ CV within 20%.

PCR data were analyzed using ABI 7500 software v.2.3 (Thermo Fisher Scientific, Waltham, MA). The results for accuracy and precision were obtained using Microsoft Excel, and the method of calculation for each value has been explained in the validation section.

### Animal experiments

Animal experiments were performed to compare the results of mtDNA-qPCR and *Alu*-qPCR in vivo. BALB/c nude mice (CAnN. Cg-Foxn1 nu/CrlOri; Orient Bio Inc., Seongnam, Republic of Korea), SD rats (Crl: CD (SD); Orient Bio Inc., Seongnam, Republic of Korea), and New Zealand white rabbits (Sam: NZW; Samtako, Republic of Korea) were grown under specific pathogen-free conditions at the Center for Laboratory Animal Research, Osong Medical Innovation Foundation. The environment of the animal facility during acclimation and experimentation was maintained as follows: temperature, 22 ± 1 °C; CO_2_ level, below 1000 ppm; relative humidity, 50% ± 10%; light time, 12 h (8:00 am to 8:00 p.m.); illumination, 150–300 Lux; ventilation frequency, 10–20 times/h. All animal experiments were conducted according to the Osong Medical Innovation Foundation guidelines for animal experimentation. All experimental protocols were approved by the Osong Medical Innovation Foundation Institutional Animal Care and Use Committee for the care and use of animals (KBIO-IACUC-2018-074). All animal experiments were conducted according to the ARRIVE guidelines. Mice were randomly allocated into control and treatment groups, and blinding was not implemented. All mice that received control and test substances were included in the analysis. The sample size for animal experiments was determined based on the specific requirements of biodistribution testing. Mice and SD rats were euthanized by placing them in a CO_2_ chamber and inducing hypoxia through gradual increase of CO_2_ gas concentration at a rate of 30 to 70%/min. New Zealand white rabbits were euthanized using Zoletil (15 mg/kg) and Xylazine (5 mg/kg). Anesthesia was induced by administering a mixed solution through the ear vein, followed by euthanasia induced by administering KCl solution (0.15 g/ml) through the ear vein at a dose of 1 ml/kg. SD rats and New Zealand white rabbits were necropsied and their livers were collected.

To compare the performances of CT product biodistribution analysis using *Alu* and mtDNA, we investigated the biodistribution of intravenously injected hADSCs (1 × 10^6^) in BALB/c nude mice; the control animals were intravenously injected with only the vehicle. Aliquots of 0.1 million hADSCs in 200 μL of ice-cold phosphate-buffered saline were intravenously injected into the tail veins of 7-week-old BALB/c nude mice (n = 6). Organs from the BALB/c nude mice (n = 3) that were not injected with hADSCs were used as NTCs. The entire brain, heart, lungs, liver, kidneys, spleen, testes, pancreas, and bone marrow were surgically removed 1 h or 7 days after injection. These tissues were rapidly frozen in liquid nitrogen and stored at − 80 °C until the extraction of genomic DNA and subsequent qPCR analysis.

### Genome extraction

The Maxwell 48 Blood Purification Kit (Promega, Madison, WI) was used to isolate gDNA. The frozen tissue samples were thawed at 4 °C for 5 h, and 25 mg of tissue was extracted using a homogenizer (Bertin Corp., Parkway Rockville, MD). DNA was then extracted from the samples according to the manufacturer’s instructions and dissolved in DNase and RNase free-H_2_O. Monkey liver DNA was purchased from AMSBIO (KG-314; Abingdon, UK). The extracted gDNA was quantified using the NanoDrop UV–Vis Spectrophotometer (Thermo Fisher Scientific, Waltham, MA) and diluted to 250 ng/μL.

### Supplementary Information

Supplementary Information.

## Data Availability

All data generated or analyzed during this study are included in this published article and its supplementary information files.
