# Supplementary material for: Mitochondrial DNA as a target for analyzing the biodistribution of cell therapy products
Source: Sci Rep. 2024 Apr 4;14:7934. doi: 10.1038/s41598-024-56591-4 (PMC10995129; doi:10.1038/s41598-024-56591-4)
Supplement: Supplementary file 1 — Supplementary Information. [file 41598_2024_56591_MOESM1_ESM.docx]

Mitochondrial DNA as a target for analyzing the biodistribution of cell therapy products

Young-Woo Cho, Jaehyeon Yoon, Suk-Gil Song, Young-Woock Noh

**Supplementary Material**

**Supplementary Tables**

**Supplementary Table S1.** Validation of *Alu* quantitative PCR and mitochondrial DNA quantitative polymerase chain reaction.

| List | | Data | Criteria | Result |
| --- | --- | --- | --- | --- |
| Calibration curve range and LLOQ | | *Alu*  NTC Ct = 28.90  STD (1.6 pg) Ct = 26.99  STD (0.32 pg) Ct = 29.33 | Accuracy: within 100% ± 15% (LLOQ: 100% ± 20%)  CV: within 15% (LLOQ: 20%)  At least five times the NTC (LLOQ) | Range  1000–1.6 pg/μg mouse  DNA |
|  |  | mtDNA  NTC Ct = 39.10  STD (0.32 pg) Ct = 32.24  STD (0.08 pg) Ct = 34.84  STD (0.16 pg) CV (%) = 39.15 |  | Range  1000–0.32 pg/μg mouse  DNA |
| LOD | | *Alu*  NTC Ct = 28.90 | Less than LLOQ  Ct value distinguished from NTC control  (NTC Ct – 2 cycle) | 1.596 pg/μg mouse  DNA |
|  |  | mtDNA  NTC Ct = 39.10 |  | 0.013 pg/μg mouse  DNA |
| Linearity | | *Alu*  R^2^: 0.997–0.999  Accuracy: 90.56–110.64 | R^2^ ≥ 0.99.  Accuracy: within 100% ± 15% (LLOQ 100% ± 20%) | PASS |
|  |  | mtDNA  R^2^ 0.998–0.999  Accuracy:  92.67%–111.53% |  | PASS |
| Accuracy  &  Precision | Intra-day repeatability  (*Alu*) | Accuracy: 93.09%–105.07%  CV: 3.05%–13.18% | Accuracy: within 100% ± 15% (LLOQ 100% ± 20%)  -CV: within 15% (LLOQ 20%) | PASS |
|  | Inter-day  repeatability  (*Alu*) | Accuracy: 86.23%–109.65%  CV: 2.76%–11.80% |  | PASS |
|  | Intra-day repeatability  (mtDNA) | Accuracy: 88.38%–99.68%  CV: 1.75%–6.67% |  | PASS |
|  | Inter-day  repeatability  (*Alu*) | Accuracy: 86.37%–100.46%  CV: 0.23%–5.35% |  | PASS |

LLOQ, lower limit of quantification; LOD, limit of detection; Ct, cycle threshold; CV, coefficient of variation; mtDNA, mitochondrial DNA.

**Supplementary Table S2**. Validation of linearity for Alu-quantitative polymerase chain reaction.

| ***Alu*** | **Assay-1** | **Assay-2** | **Assay-3** |
| --- | --- | --- | --- |
| Slope | -3.52 | -3.431 | -3.352 |
| Y-intercept | 29.818 | 29.214 | 28.634 |
| R^2^ | 0.999 | 0.998 | 0.997 |

| ***Alu*** | **Human DNA pg/μg mouse DNA** | | | | |
| --- | --- | --- | --- | --- | --- |
|  | **STD6** | **STD5** | **STD4** | **STD3** | **STD2** |
|  | 1,000.00 | 200.00 | 40.00 | 8.00 | 1.60 |
| Assay-1 | 1,015.47 | 202.02 | 37.98 | 8.37 | 1.60 |
| Accuracy (%) | 101.55 | 101.01 | 94.95 | 104.63 | 99.80 |
| Criteria [Accuracy (%)] | 100 ± 15 | 100 ± 15 | 100 ± 15 | 100 ± 15 | 100 ± 20 |
| Result | PASS | PASS | PASS | PASS | PASS |
| Assay-2 | 1,023.77 | 202.18 | 38.53 | 7.38 | 1.72 |
| Accuracy (%) | 97.68 | 98.92 | 103.81 | 108.42 | 93.28 |
| Criteria [Accuracy (%)] | 100 ± 15 | 100 ± 15 | 100 ± 15 | 100 ± 15 | 100 ± 20 |
| Result | PASS | PASS | PASS | PASS | PASS |
| Assay-3 | 1,094.49 | 183.63 | 39.76 | 7.23 | 1.77 |
| Accuracy (%) | 91.37 | 108.91 | 100.59 | 110.64 | 90.56 |
| Criteria [Accuracy (%)] | 100 ± 15 | 100 ± 15 | 100 ± 15 | 100 ± 15 | 100 ± 15 |
| Result | PASS | PASS | PASS | PASS | PASS |
| Mean | 1,044.58 | 195.94 | 38.76 | 7.66 | 1.69 |
| s.d. | 43.42 | 10.66 | 0.91 | 0.62 | 0.09 |
| Accuracy (%) | 97.21 | 103.10 | 97.99 | 109.27 | 94.32 |
| Criteria [Accuracy (%)] | 100 ± 15 | 100 ± 15 | 100 ± 15 | 100 ± 15 | 100 ± 20 |
| Result | PASS | PASS | PASS | PASS | PASS |

s.d., standard deviation

**Supplementary Table S3.** Validation of linearity for mitochondrial DNA-quantitative polymerase chain reaction.

| **mtDNA** | **Assay-1** | **Assay-2** | **Assay-3** |
| --- | --- | --- | --- |
| Slope | -3.4950 | -3.46 | -3.428 |
| Y-intercept | 31.602 | 31.092 | 31.382 |
| R^2^ | 0.999 | 0.998 | 0.998 |

| **mtDNA** | **Human DNA pg/μg mouse DNA** | | | | | |
| --- | --- | --- | --- | --- | --- | --- |
|  | **STD6** | **STD5** | **STD4** | **STD3** | **STD2** | **STD1** |
|  | 1,000.00 | 200.00 | 40.00 | 8.00 | 1.60 | 0.32 |
| Assay-1 | 975.57 | 199.75 | 40.42 | 7.96 | 1.78 | 0.30 |
| Accuracy (%) | 97.56 | 99.88 | 101.05 | 99.50 | 111.53 | 94.58 |
| Criteria [Accuracy (%)] | 100 ± 15 | 100 ± 15 | 100 ± 15 | 100 ± 15 | 100 ± 15 | 100 ± 20 |
| Result | PASS | PASS | PASS | PASS | PASS | PASS |
| Assay-2 | 966.98 | 206.20 | 39.78 | 8.11 | 1.64 | 0.31 |
| Accuracy (%) | 103.41 | 96.99 | 100.56 | 98.65 | 97.30 | 103.56 |
| Criteria [Accuracy (%)] | 100 ± 15 | 100 ± 15 | 100 ± 15 | 100 ± 15 | 100 ± 15 | 100 ± 20 |
| Result | PASS | PASS | PASS | PASS | PASS | PASS |
| Assay-3 | 1,067.67 | 203.61 | 37.33 | 7.88 | 1.51 | 0.35 |
| Accuracy (%) | 93.66 | 98.23 | 107.14 | 101.51 | 105.66 | 92.67 |
| Criteria [Accuracy (%)] | 100 ± 15 | 100 ± 15 | 100 ± 15 | 100 ± 15 | 100 ± 15 | 100 ± 20 |
| Result | PASS | PASS | PASS | PASS | PASS | PASS |
| Mean | 1,003.41 | 203.19 | 39.18 | 7.98 | 1.65 | 0.32 |
| s.d. | 55.82 | 3.25 | 1.63 | 0.12 | 0.14 | 0.02 |
| Accuracy (%) | 97.23 | 98.31 | 103.17 | 99.70 | 108.30 | 94.88 |
| Criteria [Accuracy (%)] | 100 ± 15 | 100 ± 15 | 100 ± 15 | 100 ± 15 | 100 ± 15 | 100 ± 20 |
| Result | PASS | PASS | PASS | PASS | PASS | PASS |

s.d., standard deviation

## **Supplementary Table S4.** Validation of run repeatability for Alu-quantitative polymerase chain reaction.

| ***Alu*** | **Human DNA pg/μg mouse DNA** | | | |
| --- | --- | --- | --- | --- |
|  | **HQC** | **MQC** | **LQC** | **LLOQ** |
|  | 800.00 | 20.00 | 4.80 | 1.60 |
| Assay-1 | 737.63 | 18.48 | 4.28 | 1.81 |
| Assay-2 | 708.17 | 19.42 | 4.55 | 1.79 |
| Assay-3 | 852.96 | 21.24 | 4.58 | 1.33 |
| Mean | 766.26 | 19.71 | 4.47 | 1.64 |
| S.D | 62.48 | 1.15 | 0.14 | 0.22 |
| Accuracy (%) | 95.78 | 98.57 | 93.09 | 102.47 |
| CV (%) | 8.15 | 5.81 | 3.05 | 13.52 |
| Criteria (Accuracy) | 100 ± 15 | 100 ± 15 | 100 ± 15 | 100 ± 20 |
| Criteria (CV) | ≤15 | ≤15 | ≤15 | ≤20 |
| Result | PASS | PASS | PASS | PASS |

| ***Alu*** | **Human DNA pg/μg mouse DNA** | | | |
| --- | --- | --- | --- | --- |
|  | **HQC** | **MQC** | **LQC** | **LLOQ** |
|  | 800.00 | 20.00 | 4.80 | 1.60 |
| Day 1 | 852.96 | 21.24 | 4.58 | 1.33 |
| Day 2 | 901.42 | 18.29 | 3.70 | 1.68 |
| Mean | 877.19 | 19.77 | 4.14 | 1.50 |
| s.d. | 24.23 | 1.47 | 0.44 | 0.18 |
| Accuracy (%) | 109.65 | 98.84 | 86.23 | 93.98 |
| CV (%) | 2.76 | 7.46 | 10.58 | 11.80 |
| Criteria (Accuracy) | 100 ± 15 | 100 ± 15 | 100 ± 15 | 100 ± 20 |
| Criteria (CV) | ≤15 | ≤15 | ≤15 | ≤20 |
| Result | PASS | PASS | PASS | PASS |

CV, coefficient of variation; s.d., standard deviation; HQC, high-quality control; MQC, medium-quality control; LQC, low-quality control; LLOQ, lower limit of quantification.

**Supplementary Table S5.** Validation of run repeatability for mitochondrial DNA-quantitative polymerase chain reaction.

| **mtDNA** | **Human DNA pg/μg mouse DNA** | | | |
| --- | --- | --- | --- | --- |
|  | **HQC** | **MQC** | **LQC** | **LLOQ** |
|  | 800.00 | 20.00 | 0.80 | 0.32 |
| Assay-1 | 702.25 | 20.05 | 0.75 | 0.30 |
| Assay-2 | 719.17 | 19.57 | 0.74 | 0.31 |
| Assay-3 | 732.91 | 18.25 | 0.64 | 0.35 |
| Mean | 718.11 | 19.29 | 0.71 | 0.32 |
| S.D | 12.54 | 0.76 | 0.05 | 0.02 |
| Accuracy (%) | 89.76 | 96.44 | 88.38 | 99.68 |
| CV (%) | 1.75 | 3.94 | 6.67 | 5.89 |
| Criteria (Accuracy) | 100 ± 15 | 100 ± 15 | 100 ± 15 | 100 ± 20 |
| Criteria (CV) | ≤15 | ≤15 | ≤15 | ≤20 |
| Result | PASS | PASS | PASS | PASS |

| **mtDNA** | **Human DNA pg/μg mouse DNA** | | | |
| --- | --- | --- | --- | --- |
|  | **HQC** | **MQC** | **LQC** | **LLOQ** |
|  | 800.00 | 20.00 | 0.80 | 0.32 |
| Day 1 | 702.25 | 20.05 | 0.75 | 0.30 |
| Day 2 | 679.66 | 20.14 | 0.83 | 0.32 |
| Mean | 690.96 | 20.09 | 0.79 | 0.31 |
| s.d. | 11.30 | 0.05 | 0.04 | 0.01 |
| Accuracy (%) | 86.37 | 100.46 | 98.40 | 97.29 |
| CV (%) | 1.63 | 0.23 | 5.35 | 2.78 |
| Criteria (Accuracy) | 100 ± 15 | 100 ± 15 | 100 ± 15 | 100 ± 20 |
| Criteria (CV) | ≤15 | ≤15 | ≤15 | ≤20 |
| Result | PASS | PASS | PASS | PASS |

CV, coefficient of variation; s.d., standard deviation; HQC, high-quality control; MQC, medium-quality control; LQC, low-quality control; LLOQ, lower limit of quantification.

**Supplementary Figures**


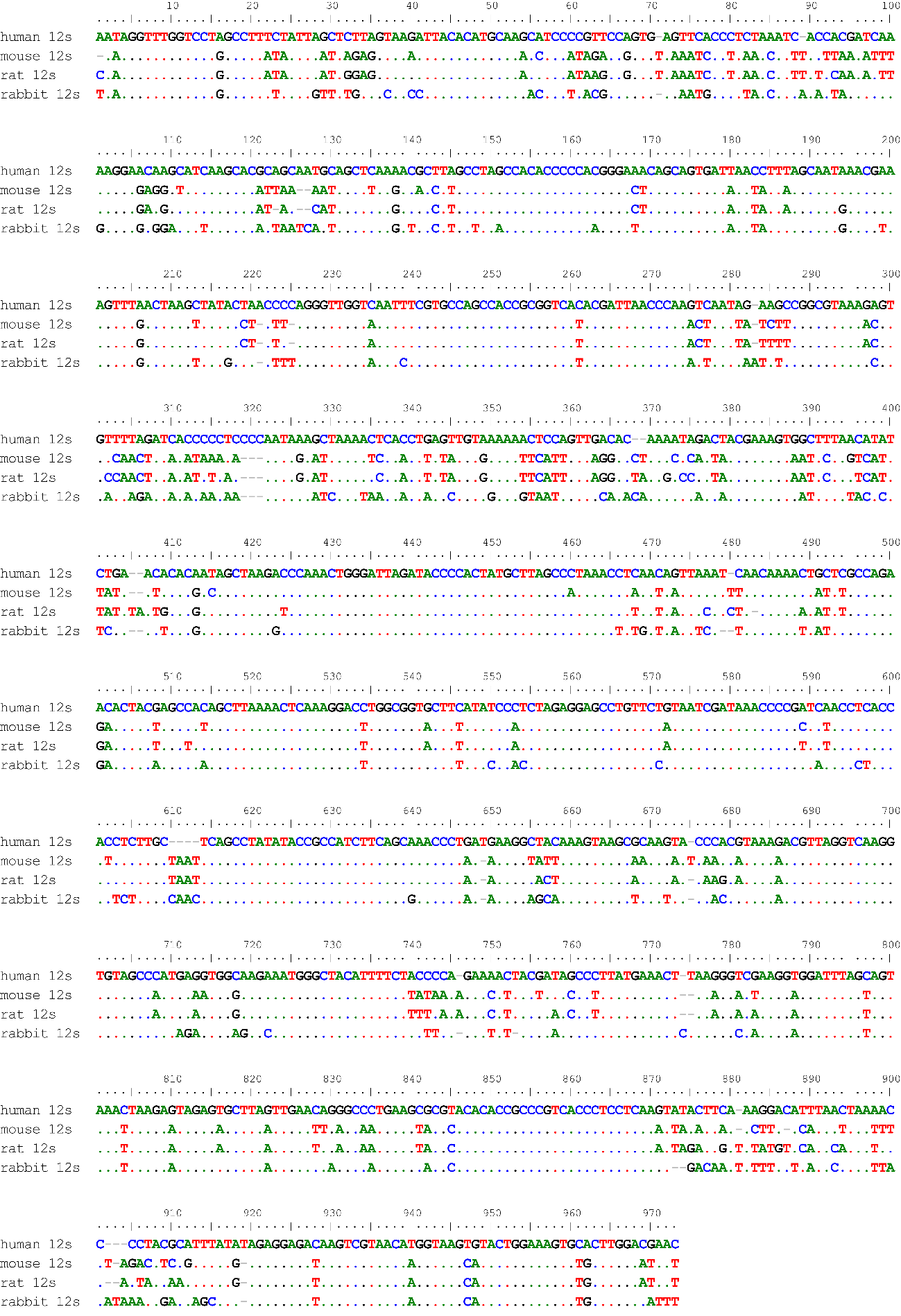


**Supplementary Fig. S1.** ClustalW results for 12S rRNA. Dots indicate sequences identical for humans and experimental animals.


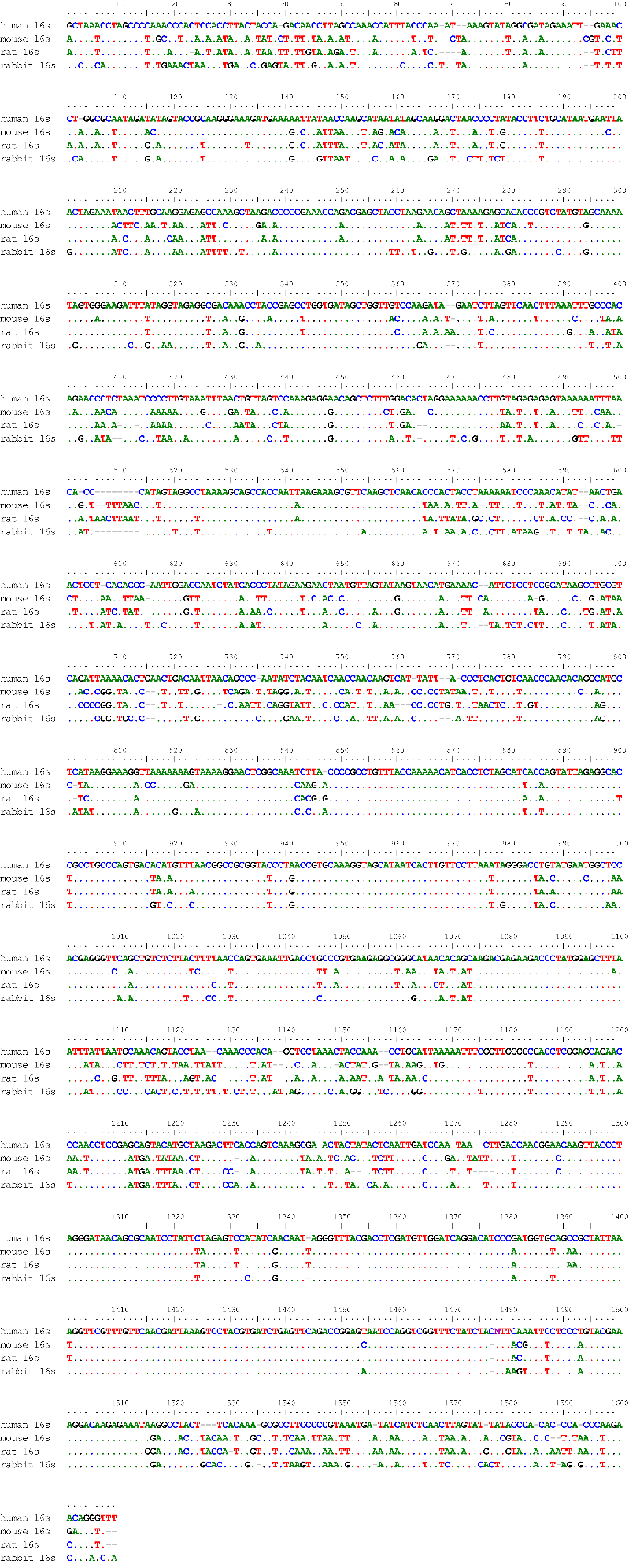


**Supplementary Fig. S2.** ClustalW results for 16S rRNA. Dots indicate sequences identical for humans and experimental animals.


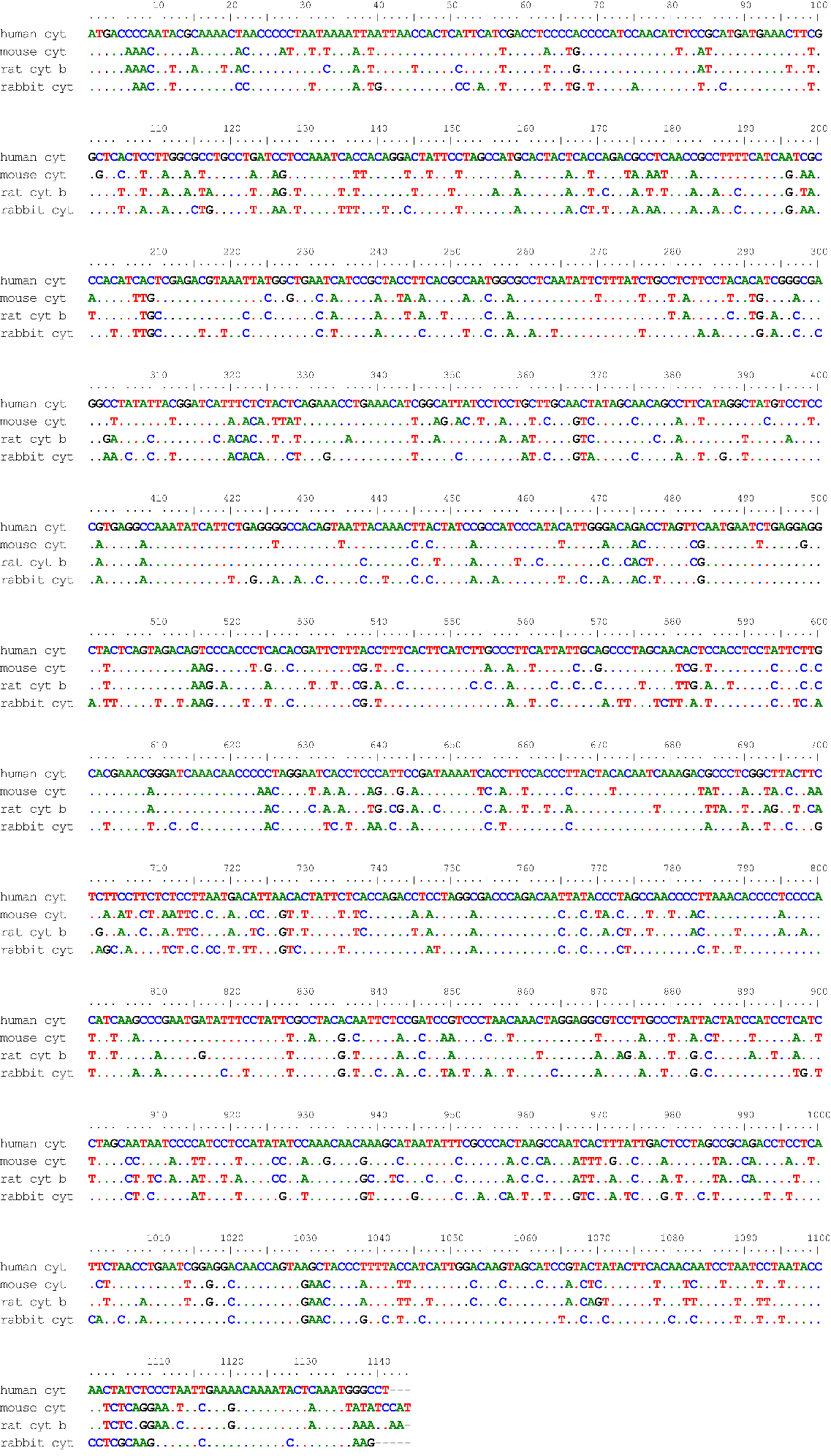


**Supplementary Fig. S3**. ClustalW results for *CYTB*. Dots indicate sequences identical for humans and experimental animals.

**
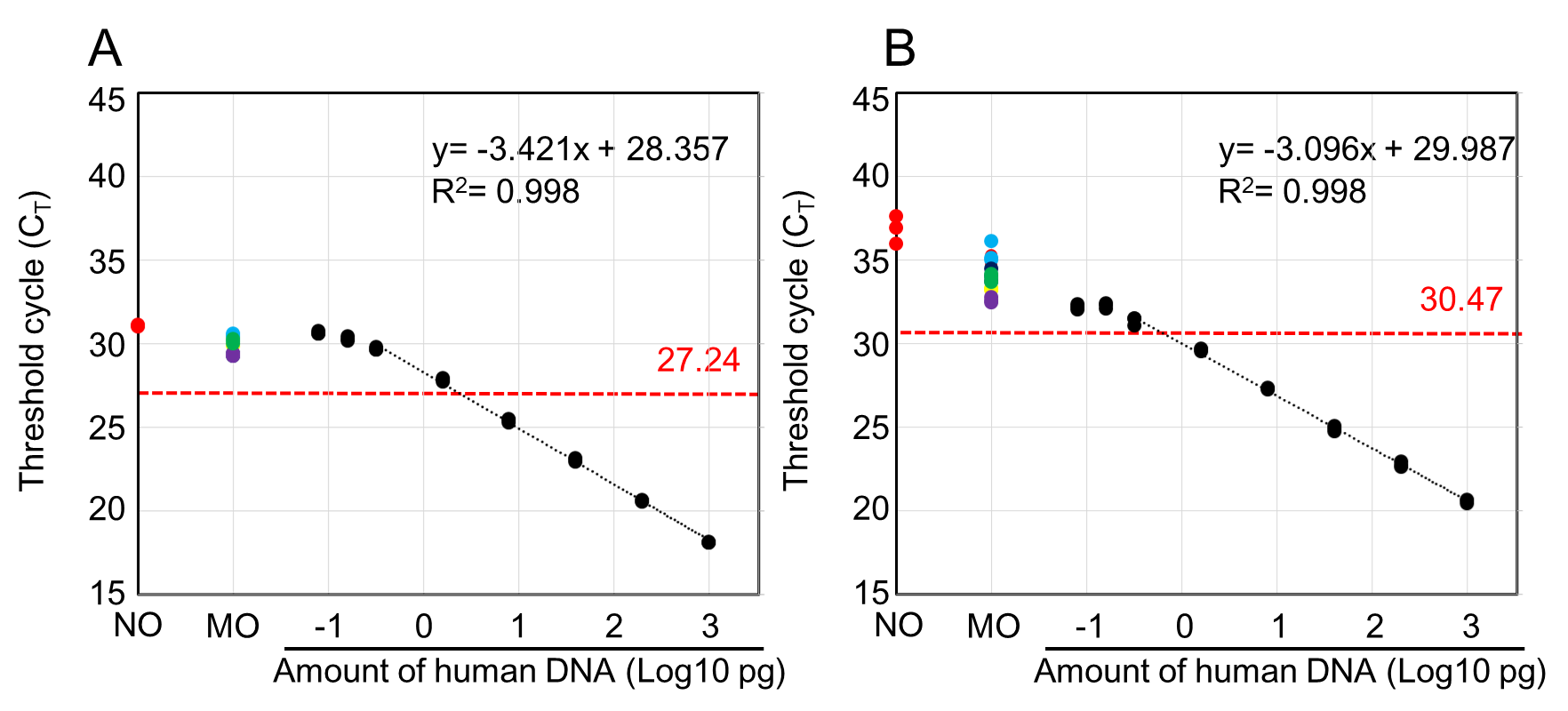
**

**Supplementary Fig. S4**. Standard curve for detecting human gDNA using primer and probe sets for *McBride et al.* **(A)**  and Funakoshi et al.. qPCR was performed in triplicate in a single run, and mean Ct values were plotted. Negative control samples contained mouse gDNA only (brain, yellow dot; heart, green dot; lung, blue hollow circle; liver, orange dot; kidney, purple dot; spleen, red hollow circle; and pancreas, blue dot) and were used as noDNA (red dots). Detection threshold was established as two cycles below the lower Ct value of the mouse DNA or noDNA (indicated by the red and dotted lines), and the corresponding number of cycles is highlighted in red. The linear approximation equations (black lines) and their corresponding R^2^ values are also displayed. gDNA, genomic DNA; mtDNA, mitochondrial DNA; qPCR, quantitative polymerase chain reaction; MO, mouse DNA; NO, noDNA.
